# Levels of Gene Expression of Immunological Biomarkers in Peri-Implant and Periodontal Tissues

**DOI:** 10.3390/ijerph17239100

**Published:** 2020-12-06

**Authors:** Luciene Cristina Figueiredo, Bruno Bueno-Silva, Cristiana Fernandes Plutarco Nogueira, Leonardo Carneiro Valadares, Katia Marina Morilla Garcia, Givelton Coimbra da Luz Filho, Luciano Milanello, Felipe Machado Esteves, Jamil Awad Shibli, Tamires Szeremeske Miranda

**Affiliations:** Dental Research Division, Guarulhos University, São Paulo 07023-070, Brazil; lucienedefigueiredo@gmail.com (L.C.F.); cris.odonto@outlook.com (C.F.P.N.); leonardocvaladares@yahoo.com.br (L.C.V.); kat_siv@hotmail.com (K.M.M.G.); supertim_7@hotmail.com (G.C.d.L.F.); lucianomilanello@gmail.com (L.M.); felipeesteves25@hotmail.com (F.M.E.); jshibli@ung.br (J.A.S.); tami.szeremeske@gmail.com (T.S.M.)

**Keywords:** periodontitis, peri-implantitis, biomarkers

## Abstract

This study compared the gene expression of the immunoinflammatory markers interleukin (IL)-6, IL-1ß, and tumor necrosis factor alpha (TNF-α), the matrix metalloproteinases (MMP)-1, -2, -8, and -9, and the tissue inhibitors of matrix metalloproteases (TIMP)-1 and -2 in the gingival tissue of individuals with periodontal and peri-implant disease. The study population included individuals with four periodontal statuses: periodontal health (PH group, n = 20); periodontitis (P group, n = 20); peri-implant health (PIH group, n = 20), and peri-implantitis (PI group, n = 20). Gingival biopsies were collected from one tooth per patient according to the inclusion criteria of each group. The mRNA levels of IL-6, IL-1ß, TNF-α, MMP-1, MMP-2, MMP-8, MMP-9, TIMP-1, and TIMP-2 were evaluated by qPCR. The levels of IL-1ß were significantly higher in the PI group when compared to the other groups (*p* < 0.05), while the levels of IL-6 were significantly higher in the groups with periodontal and peri-implant disease when compared with the healthy groups (*p* < 0.05); however, the levels of IL-6 did not differ between the PI and P groups (*p* > 0.05). For all other studied biomarkers, no significant differences were observed between groups (*p* > 0.05). IL-6 and IL-1ß presented higher levels of mRNA in diseased periodontal and peri-implant tissues. However, the expression of metalloproteinases and their inhibitors did not differ between the different periodontal statuses.

## 1. Introduction

In recent decades, dental implants have been used successfully to rehabilitate, both partially and completely, edentulous patients. However, the occurrence of implant loss may be closely related to the presence of peri-implant bacterial infection. Peri-implantitis is defined as a biofilm-associated pathologic condition occurring in the tissue around dental implants, characterized by inflammation and subsequent progressive loss of the supporting bone. Although the formation of bacterial biofilm can be considered an important first step in the progression of peri-implant disease, the immune-–inflammatory response generated by bacterial stimuli is responsible for the tissue damage associated with peri-implantitis [1,2,3,4].

The components of bacterial biofilm, such as lipopolysaccharides (LPS) and toxins, initiate the immune–inflammatory response by activating the host’s defense cells, which include polymorphonuclear cells (PMNs), thus triggering a response against the microbial invasion. The defense cells’ activation results in the production of inflammatory mediators such as cytokines, chemokines, prostaglandins, and proteolytic enzymes that, in turn, alter the connective tissue and bone metabolism [5,6]. The defense process against a bacterial infection is initially mediated by pro-inflammatory cytokines, such as tumor necrosis factor alpha (TNF-α), interleukin (IL)-1, IL-6, IL-8, and IL-12, in addition to monocyte/lymphocyte infiltration [7,8,9].

An implant placement results in the healing process, which involves soft and hard tissues around the surgical area. Inflammatory cells are recruited as a response to this trauma, playing a role in healing wounds or prolonging the inflammatory response by favoring, delaying, or even preventing implant osseointegration [10]. However, after successful osseointegration, inflammatory processes may occur around the implants, resulting in mucositis or peri-implantitis [11,12].

Although several studies have analyzed the influence of inflammatory mediators in the etiopathogenesis of peri-implantitis, so far, no research has compared clinical situations of health and disease (periodontal and peri-implant). Considering that, currently, there are no effective therapeutic strategies against peri-implantitis, further investigation of the molecular pathophysiology of peri-implantitis is necessary to provide new effective treatment options and also to better understand the possible biomarkers capable of contributing to the diagnosis of peri-implant diseases. The null hypothesis of this study was to verify that there are differences in the expression of immunoinflammatory biomarkers between periodontal and peri-implant diseases. In addition, this study sought to assess the differences between healthy and diseased tissues.

Thus, the aim of this investigation was to compare the gene expression of immunoinflammatory markers (IL-6, IL-1ß, and TNF-α), the matrix metalloproteinases (MMP)-1, -2, -8, and -9, and the tissue inhibitors of matrix metalloproteases (TIMP)-1 and -2 in the gingival tissue of individuals with periodontal and peri-implant disease.

## 2. Materials and Methods

### 2.1. Study Population

Adult subjects were selected (>35 years old), male or female, with generalized periodontitis, periodontal health, peri-implantitis, or peri-implant health from the population seeking dental treatment at the University of Guarulhos (Guarulhos, SP, Brazil) and were distributed into one of the following groups:

- PH group (n = 20): individuals with periodontal health;
- P group (n = 20): individuals with periodontitis;
- PIH group (n = 20): individuals with peri-implant health;
- PI group (n = 20): individuals with peri-implantitis.

All eligible individuals were invited to participate in the study and were thoroughly informed of its nature, potential risks, and benefits. Participants who agreed to participate in the study signed a free and informed consent form (ICF), answered an anamnesis questionnaire, and received periodontal and peri-implant therapy free of charge, in accordance with the guidelines and rules of the National Health Council (Resolution No. 466/2012). The study was conducted in accordance with the Declaration of Helsinki, and the protocol was approved by the Human Research Ethics Committee at the University of Guarulhos.

### 2.2. Exclusion and Inclusion Criteria

General exclusion criteria (considered for all groups):

- Use of antibiotics in the 6 months preceding the study;
- Use of corticosteroids, non-steroidal anti-inflammatory drugs, immunosuppressants, estrogen receptor modulators, or medications that can influence bone metabolism (alendronate, calcitonin, and others) during the 6 months prior to the study;
- Smoker or ex-smoker (previous 5 years);
- Individuals with diabetes, osteoporosis, immune disorders, hepatitis, or other systemic disorders that could compromise the individual’s defense response;
- Pregnant or lactating.

Specific inclusion criteria:

For individuals with peri-implantitis:

- Bleeding on probing and/or suppuration, with a probing depth (PD) >4 mm; radiographic bone loss greater than 3 mm, with at least 50% of peri-implant bone remaining (otherwise, implant was considered lost);
- Presenting at least one implant-supported prosthesis, using smooth-surface implants (commercially pure titanium), external hexagon and threadable, under function for at least 1 year, affected by peri-implantitis (if the individual had more than one implant affected by peri-implantitis, only one implant was evaluated);
- The absence of mobility, breakage of screws, or loosening of prosthetic components next to implant-supported prostheses, in order to minimize the influence of possible occlusal trauma.

For individuals with periodontitis, those who initially met the criteria indicated below, but after conventional periodontal treatment of scaling and root planing, remained with sites with PD ≥5 mm and with bleeding on probing (BoP):

- Minimum 15 teeth, excluding third molars and teeth indicated for extraction;
- Those with periodontitis stage III or IV, grade B or C, presenting at least 30% of the sites having PD and a clinical attachment level (CAL) ≥5 mm.

For clinically healthy individuals:

- Selected individuals with a PD and CAL ≤3 mm at all sites, without BoP, with indication for reconstructive/corrective surgery (PIH group) and gingivectomy or increased clinical crown (PH group).

### 2.3. Clinical and Radiographic Examination

The peri-implant and periodontal examination was performed by a previously trained and calibrated examiner. The measures were determined using a North Carolina manual periodontal probe (PCP UNC 15 Hu-Friedy Mfg Co Inc., Chicago, IL, USA). Measurements were made on 6 faces of the implant or tooth: mesio-vestibular, mid-buccal, distal–buccal, mesio-lingual, mid-lingual, and distolingual. The methodology used for the calibration for the measurement performed on both the remaining teeth and the osseointegrated implants was recommended by Araújo et al. (2003) [13], in which the standard error of the measure (s.e.m.) and the average percentage error (a.p.e.) were evaluated for the continuous clinical parameters (PD and CAL). The s.e.m.. and a.p.e. intra-examiner values obtained for the remaining teeth were 0.22 mm/5.2% for the PD and 0.30 mm/6.33% for the CAL, respectively. The s.e.m.. and a.p.e.. intra-examiner values obtained for the implants were 0.09 mm and 2.2% for the probing depth and 0.08 mm and 3.99% for the clinical level of insertion. Categorical variables considering only the presence or absence of the clinical parameter were obtained for teeth and implants with an average level of agreement determined by the kappa light test in the value of 89%, as follows:

- Presence (1) or absence (0) of a visible bacterial biofilm;
- Presence (1) or absence (0) of marginal bleeding (MB);
- Presence (1) or absence (0) of bleeding on probing;
- Presence (1) or absence (0) of suppuration (SUP);
- PD (mm)—characterized by the distance from the peri-implant margin or gingival margin to the bottom of the bag;
- CAL (mm)—characterized by the distance from a fixed point previously determined (connector/implant junction or bar in cases of overdentures or cement–enamel junction) to the bottom of the bag.

Radiographic examinations were performed using Ektaspeed films (Kodak, Eastman, CO, USA) and using positioners for the parallelism technique adapted for each patient with the help of condensation silicone. The radiographs obtained were processed by the time–temperature method and then digitized using a digital camera (Canon EOS 300D, Tokyo, Japan). To obtain vertical bone loss, the distance between the prosthetic connector and the peri-implant alveolar bone crest was measured using the Image Tool 3.0 software (http://ddsdx.uthscsa.edu/dig/itdesc.html). These measurements were made by a single examiner who was previously trained and were used to complement the diagnosis of peri-implantitis.

### 2.4. Gingival Tissue Sampling and Gene Expression Analysis

Gingival biopsies with junctional and sulcular epithelium and connective tissue were harvested from one tooth per patient according to the inclusion criteria for each group. The gingival tissues were later stored in RNA (Ambion Inc., Austin, TX, USA) at −80 °C for gene expression [14].

RNA extraction, cDNA synthesis, and PCR reactions were performed, as previously described [13]. The mRNA levels of IL-1ß, IL-6, TNF-α, MMP-1, MMP-2, MMP-8, MMP-9, TIMP-1, TIMP-2, and glyceraldehyde-3-phosphate dehydrogenase (GAPDH) were analyzed in real time by PCR. The amplification profiles, primer sequences, and lengths of PCR products are presented in Table 1 [14,15]. The results were taken as the relative amounts of the target gene, using GAPDH as the internal reference gene by means of a relative quantification tool (LightCycler Software 4, Roche Diagnostics GmbH, Mannheim, Germany).

### 2.5. Statistical Analysis

A post-hoc analysis was conducted to determine the power of the analyses presented in this study. Considering differences of at least 2.2 for the mRNA levels of IL-6 (primary outcome variable) between the PH and P groups and a standard deviation of 3.9, it was determined that 20 subjects per group would provide 95% power with an alpha of 0.05.

The mean mRNA levels of the biomarkers in the gingival tissues were computed for each subject and, subsequently, across groups. Data were examined for normality by the Shapiro–Wilk test and non-parametric methods were used for data that did not achieve normal distribution. The significance of differences for age and clinical parameters were analyzed using one-way ANOVA and the Tukey test, while mRNA levels were compared using the Kruskal–Wallis test, followed by post-hoc analyses using the Dunn test. The chi-square test compared the differences between genders. The level of significance for all analyses was set at 5%.

## 3. Results

This study was conducted between March 2018 and July 2020. The studied population was composed of 80 individuals selected out of nearly 600 who were screened. Thirty-seven individuals were men and 43 were women, which was considered homogeneous in relation to the frequency of the genders in the groups (*p* > 0.05). The average age of the study participants was 43.12 ± 5.02 years. No statistical differences were found for the age of individuals in groups PH (41.6 ± 4.4 years), P (43.2 ± 7.1 years), PIH (42.7 ± 4.3 years), and PI (44.8 ± 3.9 years) (*p* > 0.05).

The data of the four groups evaluated are shown in Table 2.

Table 3 presents the mRNA levels of all biomarkers. The levels of IL-1 ß were significantly higher in the PI group compared to the other groups (*p* < 0.05), while the levels of IL-6 were significantly higher in the groups with periodontal and peri-implant disease when compared to the healthy groups (*p* < 0.05). However, the levels of IL-6 did not differ between the PI and P groups (*p* > 0.05). For all of the other biomarkers studied (TNF-α, MMP-1, MMP-2, MMP-8, MMP-9, TIMP-1, and TIMP-2), no significant differences were observed between the groups (*p* > 0.05).

## 4. Discussion

This study evaluated the gene expression of IL-1ß, IL-6, TNF-α, MMP-1, MMP-2, MMP-8, MMP-9, TIMP-1, and TIMP-2 in the gingival tissue of individuals with periodontitis or peri-implantitis compared to individuals without periodontal or peri-implant disease. Overall, the results indicated that the mRNA levels of IL-1ß and IL-6 were increased in diseased tissues when compared to healthy tissues. However, the levels of both markers did not differ between the periodontal and peri-implant tissues. For the other biomarkers, no significant differences were observed between the studied groups.

Both periodontal and peri-implant disease are similar in etiologic and pathogenic aspects [16,17,18]. IL-1β has an essential role in inducing the inflammatory and immunological response to antigens associated with increased levels of prostaglandins, metalloproteinases, pro-inflammatory cytokines such as IL-6 and TNF-α, and biomarkers related to the osteoclastogenesis process [19]. Studies have shown that both IL-1ß and IL-6 are very important proinflammatory interleukins involved in the pathogenesis of infectious diseases [19,20]. Corroborating our results, previous studies have shown that the levels of IL-1ß and IL-6 are increased in diseased periodontal and peri-implant tissues [20,21].

MMPs belong to the group of proteases, an enzymes that catalyze the breaking of peptide bonds in proteins and have been associated with the degradation of almost all components of the extracellular membrane and the basement membrane [22]. The balance of MMP activity is controlled mainly by TIMPs [23,24]. Several studies have demonstrated the involvement of these enzymes and their inhibitors in the damage of periodontal tissues [25,26,27,28,29]. However, regarding peri-implant tissues, it is important to note that there is little scientific information published on this subject and the results are contradictory. Several studies have evaluated these same biomarkers in different types of samples using different methodologies, which makes it difficult to compare between studies, as the detection in mRNA or protein levels do not always coincide.

In general, studies have shown that the levels of MMP-1, -2, -8, and -9, are increased in diseased periodontal and peri-implant tissues when compared to healthy tissues [30,31,32,33,34,35]. However, according to our results, other studies have not observed significant differences in these proteases between healthy and diseased tissues [36,37,38]. Recently, Ghassib et al. (2019) [21], in a systematic review, compiled data from nineteen studies that assessed the levels of IL-1β, IL-6, TNF-α, and/or matrix MMP-8 in the crevicular fluid of healthy implants, peri-implant mucositis, and peri-implantitis. According to the results, IL-1β, IL-6, and TNF-α, levels were significantly higher in mucositis and peri-implantitis than in healthy implants. However, studies evaluating MMP-8 have been limited and controversial, and a meta-analysis cannot be performed [21]. Kensara et al. (2020) [39] also evaluated the existing evidence on the immune response associated with peri-implantitis in comparison to healthy implants; according to the results, the pro-inflammatory cytokines and MMPs (IL-1β, IL-6, IL-17, TNF-α, MMP-2, and MMP-9) are expressed at a higher level in peri-implantitis sites compared to in the control group.

As for the mRNA levels, this study was the first to evaluate the gene expression of three main immunoinflammatory markers and the MMPs in the gingival tissue of individuals with periodontal and peri-implant disease compared to individuals without disease. In addition, the four groups studied made it possible to compare periodontal and peri-implant tissues. This study has limitations, however; due to its cross-sectional nature, the effect of the altered levels of IL-1 β and IL-6 over the long term was missed. Unexpectedly, no significant differences were observed for MMPs, possibly due to the limited number of samples studied.

## 5. Conclusions

In conclusion, IL-6 and IL-1ß presented higher levels of mRNA in diseased periodontal and peri-implant tissues. However, in this study, the expression of metalloproteinases and their inhibitors did not differ among groups.

## Figures and Tables

**Table 1:** Primer sequences, amplification profile and estimated length of the PCR product for each biomarker [14,15].

Biomarkers	Sequence	Amplification Profile (Temperature (°C)/Time (s))	Length of PCR Product (bp)
IL-6	5′ GCAGGACATGACAACTCATC 3′ AAGTTCTGTGCCCAGTGG	95/10, 56/5, 72/6	159
IL-1ß	5′ CACCTTCTTTCCCTTCATCTTTG 3′ GCTTTCAGTTCATATGGACCAG	95/10, 56/5, 72/7	158
TNF-α	5′ CCAGGGACCTCTCTCTAATCA 3′ CTGGTTATCTCTCAGCTCCAC	95/10, 56/5, 72/7	178
MMP-1	5′ AGCTGCTTACGAATTTGCC 3′ GCAGCATCGATATGCTTCAC	95/10, 55/10, 72/7	136
MMP-2	5′ CGCAGATGCCTGGAATG 3′ GTCGGATTTGATGCTTCCAAAC	95/10, 55/10, 72/7	151
MMP-8	5′ CTAGACAGTACCCTTGGCC 3′ CTGCAAAGACCCTGGTAAG	95/10, 55/10, 72/7	154
MMP-9	5′ GCTACCACCTCGAACTTTGAC 3′ CTCAGTGAAGCGGTACATAGG	95/10, 56/10, 72/7	162
TIMP-1	5′ CAGACCACCTTATACCAGCG 3′ CCAGCAATGAGAAACTCCTC	95/10, 55/10, 72/7	145
TIMP-2	5′ CTGGGAGGGTATCCAGGAATC 3′ CACCATACAGGAAGCGAAC	95/10, 56/10, 72/7	169
GAPDH	5′ CTGAGTACGTCGTGGAGTC 3′ TGATGATCTTGAGGCTGTTGTC	95/10, 56/10, 72/7	187

IL: interleukin; TNF: tumor necrosis factor; MMP: matrix metalloproteinase; TIMP-1: tissue inhibitor of matrix metalloprotease; TIMP-2: tissue inhibitor of metalloproteinases; GAPDH: glyceraldehyde-3-phosphate dehydrogenase.

**Table 2:** Demographic characteristics and mean periodontal clinical parameters (± SD) of the study population.

Parameters	Groups			
	PH	P	PIH	PI
Gender (M/F) *	09/11	10/10	10/10	8/12
Age (years)	41.6 ± 4.4	43.2 ± 7.1	42.7 ± 4.3	44.8 ± 3.9
% of sites with visible plaque	31.7 ± 12.8 ^A^	57.5 ± 12.3 ^B^	34.2 ± 13.7 ^A^	50.3 ± 10.8 ^B^
% of sites with MB	7.3 ± 2.8 ^A^	51.8 ± 8.3 ^B^	5.2 ± 2.2 ^A^	48.7 ± 9.1 ^B^
% of sites with BoP	14.7 ± 5.5 ^A^	72.9 ± 16.1 ^B^	12.1 ±5.1 ^A^	82.3 ± 17.3 ^B^
PD (mm)	2.2 ± 0.3 ^A^	3.74 ± 0.7 ^B^	2.6 ± 0.8 ^A^	5.5 ± 1.2 ^B^
CAL (mm)	2.3 ± 0.4 ^A^	4.23 ± 0.8 ^B^	2.7 ± 0.3 ^A^	5.8 ± 1.3 ^B^
% of sites with suppuration	0.00 ± 0.0 ^A^	2.7 ± 0.9 ^B^	0.00 ± 0.0 ^A^	3.1 ± 0.1 ^B^

* Chi-square test (*p* > 0.05). Different letters (^A, B^) indicate significant differences between groups (one-way ANOVA and the Tukey test; *p* < 0.05). M/F: male/female; MB: marginal bleeding; BoP: bleeding on probing; PD: probing depth; CAL: clinical attachment level; PH: periodontal health; P: periodontitis; PIH: peri-implant health; PI: peri-implantitis.

**Table 3:** Mean mRNA levels (± SD) for all biomarkers studied.

Biomarkers	Groups			
	PH	P	PIH	PI
IL-1ß	0.0 ± 0.0 ^B^	0.0 ± 0.01 ^B^	0.0 ± 0.0 ^B^	0.2 ± 0.4 ^A^
IL-6	0.1 ± 0.2 ^A^	2.3 ± 4.1 ^B^	0.2 ± 0.1 ^A^	7.8 ± 1.7 ^B^
TNF-α	0.1 ± 0.2	1.9 ± 4.7	0.0 ± 0.1	1.3 ± 3.9
MMP-1	1.4 ± 4.3	1.30 + 0.10	0.90 + 0.10	1.20 + 0.20
MMP-2	4.7 ± 0.3	5.9 + 0.1	4.9 + 0.1	6.2 + 0.2
MMP-8	0.2 ± 0.1	0.4 ± 0.1	0.1 + 0.1	0.3 + 0.2
MMP-9	0.6 ± 0.1	3.4 ± 0.9	2.3 ± 5.3	3.7 ± 0.6
TIMP-1	6.8 + 0.1	8.6 + 0.1	6.6 + 0.2	8.1 + 0.3
TIMP-2	0.7 ± 0.1	2.0 ± 5.30	0.5 + 0.2	1.3 ± 0.3

Different letters (^A, B^) indicate significant differences between groups (Kruskal–Wallis and Dunn tests; *p* < 0.05). IL: interleukin; TNF: tumor necrosis factor; MMP: matrix metalloproteinases; TIMP: inhibitor of metalloproteinase; SD: standard deviation.
